# Nitric oxide in sepsis: pathophysiological mechanisms and therapeutic implications

**DOI:** 10.1515/biol-2025-1315

**Published:** 2026-05-04

**Authors:** Jun Chen, Zhenghang Li, Zhuo Li, Zhaoqun Zhou

**Affiliations:** Department of Emergency/Critical Care Medicine, Children’s Hospital of Nanjing Medical University, No. 72 GuangZhou Road, Nanjing, Jiangsu Province, 210008, China

**Keywords:** immunomodulation, nitric oxide, pathogenesis, sepsis

## Abstract

Sepsis is a life-threatening syndrome defined by organ dysfunction arising from a dysregulated host response to infection. In its more severe forms, it may progress to multiple organ failure and is associated with a substantial increase in mortality risk. Nitric oxide (NO), a gaseous signaling mediator, has attracted attention because of its context-dependent roles in both physiological regulation and pathological processes. NO participates in intracellular signaling pathways and modulates protein function through post-translational mechanisms. In patients with sepsis, altered NO production has been closely linked to circulatory shock, vascular hyporeactivity, and multiple organ dysfunction, with particularly significant effects on cardiovascular and renal systems. Despite extensive investigation, the precise role of NO at different stages of sepsis remains incompletely defined. Clarifying the temporal and mechanistic aspects of NO dysregulation may help determine its utility as a biomarker and as a potential therapeutic target. Future clinical strategies should focus on modulating excessive NO activity without compromising its essential physiological functions, thereby improving the translational feasibility and clinical applicability of NO-directed interventions.

## 1 Introduction

Sepsis is defined as life-threatening organ dysfunction caused by a dysregulated host response to infection [1]. It is associated with substantial morbidity and mortality and represents a major global cause of patient mortality. Approximately half of sepsis cases progress to septic shock, which is characterized by refractory hypotension and multiple organ failure. Severe sepsis and septic shock are strongly correlated with mortality rates of 20–30 % [2]. Hemodynamic, macrocirculatory, and microcirculatory failure represent key features of severe sepsis and septic shock.

Nitric oxide (NO), a gaseous signaling molecule, is an important biochemical product in mammalian cells and exerts diverse physiological and pathological effects. Numerous systemic and local mediators act through NO-dependent pathways, underscoring its importance in biomedical research. Given its role as a fundamental cellular messenger and mediator, this review examines the function of NO in sepsis and highlights recent advances in understanding its complex pathophysiological mechanisms.

Prior to 2015, multiple reviews summarized the role of NO in sepsis. With the continued evolution of concepts and understanding of sepsis among researchers and clinicians worldwide, the present review aims to further summarize recent advances in this field, focusing on key terms including NO, nitric oxide synthase (NOS), sepsis, and septic shock.

## 2 Origin and biosynthesis of NO

NO is a small inorganic molecule that readily diffuses across the cytoplasmic membrane. It is biologically unstable, with a half-life of less than 6 s, and is rapidly converted to its more stable metabolites, nitrate and nitrite, upon interaction with oxygen or reactive oxygen species such as superoxide. NO is synthesized by a family of enzymes known as nitric oxide synthases (NOS), which catalyze the oxidation of the guanidinium nitrogen of l-arginine, generating l-citrulline as a by-product.

To date, three NOS isoforms have been identified, purified, and cloned. These isoforms differ in subcellular localization, amino acid sequence, regulatory mechanisms, and functional roles. NOS is classified into endothelial nitric oxide synthase (eNOS), neuronal nitric oxide synthase (nNOS), and inducible nitric oxide synthase (iNOS). Both eNOS and nNOS are constitutively expressed and are collectively termed constitutive NOS (cNOS) [3]. These enzymes are continuously present and can be rapidly activated to produce small amounts of NO, thereby contributing to physiological regulatory processes in multiple tissues. In contrast, iNOS was first identified in macrophages and can be induced in nearly all mammalian tissues. Once activated, iNOS catalyzes the sustained production of large quantities of NO.

## 3 Role and mechanisms of NO in sepsis

### 3.1 NO and septic shock

Organ dysfunction and failure observed in sepsis are closely associated with the biological effects of NO. At the cellular level, NO modulates enzymatic activity, resulting in either activation or inhibition depending on the molecular target. The physiological consequences of these interactions depend on both the site of NO production and the quantity generated. Small amounts of NO produced by cNOS isoforms activate NO-sensitive enzymes within the same cell or in adjacent cells. Owing to its short half-life, NO primarily mediates autocrine and paracrine signaling; rapid degradation in oxygenated environments and binding to hemoglobin limit systemic endocrine effects.

When iNOS is expressed, substantially larger amounts of NO are produced. In this setting, NO not only activates NO-sensitive enzymes but also binds to iron-containing enzymes, leading to their functional inhibition [4], 5].

Within endothelial cells, NO diffuses into neighboring vascular smooth muscle cells, where it binds to the ferrous ion in the heme moiety of soluble guanylate cyclase (sGC). This interaction activates sGC and increases intracellular cyclic guanosine monophosphate (cGMP) levels. Elevated cGMP reduces calcium influx into vascular smooth muscle cells, thereby promoting smooth muscle relaxation and vasodilation [6], 7]. Inhibition of NO synthesis using competitive inhibitors such as NG-monomethyl-l-arginine abolishes the tonic vasodilatory effect mediated by eNOS and results in increased resting blood pressure [8].

In rodent models [9], 10], endothelial cells express iNOS in response to tumor necrosis factor (TNF) and interferon-γ (IFN-γ). Vascular smooth muscle cells in both humans and rodents express iNOS following stimulation with interleukin-1 (IL-1), TNF, IFN-γ, or lipopolysaccharide (LPS). Excessive iNOS expression within the vascular wall is considered a major contributor to the profound vasodilation characteristic of septic shock. Clinical studies evaluating L-NMMA have shown that NOS inhibition can reverse hypotension in septic shock; however, reductions in cardiac output may limit its clinical applicability [11].

In a porcine endotoxemia model, the selective iNOS inhibitor N-[3-(aminomethyl)benzyl]acetamidine (1,400 W) restored intestinal mucosal microcirculatory perfusion [12]. More recently, treatment with the selective iNOS inhibitor 1,400 W during sepsis was shown to prevent vascular hyporesponsiveness and preserve α1-adrenergic receptor density [13]. These findings indicate that suppression of excessive NO production via iNOS may restore systemic blood pressure in patients with sepsis. Cytokine- and LPS-induced iNOS expression leads to vasorelaxation that is resistant to vasoconstrictors; this effect may be attenuated by glucocorticoids or NOS inhibitors. The degree of NO production during endotoxin shock correlates with the severity of hypotension observed clinically. Thus, activation of the iNOS–NO pathway plays a central role in vasodilation and vasopressor resistance in septic shock.

Activation of sGC also represents a key mechanism through which NO inhibits platelet adhesion and aggregation [14]. NO acts synergistically with prostacyclin, which increases intracellular cyclic adenosine monophosphate (cAMP) in platelets to suppress aggregation. Unlike prostacyclin, NO not only inhibits platelet aggregation but also reduces platelet adhesion to endothelial cells. Platelets themselves can synthesize NO, establishing a negative feedback mechanism that limits activation. These effects may help prevent microvascular thrombosis during sepsis-associated hypotension and contribute to cytoprotection during tissue hypoperfusion. Enhancement of eNOS activity in platelets and endothelial cells may reduce neutrophil–endothelial interactions and support maintenance of microvascular patency in septic shock [15].

NO also modulates leukocyte adhesion to endothelial cells. Together with superoxide, NO influences mast cell activation and degranulation, processes that promote leukocyte adhesion. Inhibition of NO synthesis, accompanied by reduced superoxide levels, decreases mast cell activation, limits the release of pro-inflammatory mediators, and reduces leukocyte adhesion to endothelial cells [16].

In summary, the iNOS–NO pathway represents a central mechanism in the vascular pathology of septic shock. Therapeutic strategies targeting this pathway must balance hemodynamic stabilization with preservation of the physiological functions of NO, including antimicrobial activity and inhibition of platelet aggregation. The clinical utility of iNOS inhibitors remains constrained by the dual role of NO, organ-specific responses, and the dynamic progression of sepsis. Future advances may depend on the development of highly selective iNOS inhibitors, biomarker-guided patient stratification (e.g., circulating LPS levels or myocardial iNOS activity), and combination targeted therapies. Continued investigation into selective iNOS inhibitors, NO scavengers, and sGC modulators may allow more precise therapeutic intervention.

### 3.2 Mechanisms of NO in sepsis-associated cardiac dysfunction

The heart is among the organs most susceptible to injury during sepsis, and severe cases may be complicated by arrhythmias and heart failure. Sepsis-induced cardiomyopathy (SICM), also termed sepsis-induced myocardial dysfunction (SIMD), is increasingly recognized as a reversible form of cardiac dysfunction occurring in patients with sepsis. In 1984, Parker et al. reported that reversible myocardial depression associated with sepsis and septic shock contributes to the development of SICM [17].

Recent evidence suggests that the pathogenesis of SICM involves several key mediators, including endotoxins, proinflammatory cytokines, and NO. Endotoxins impair myocardial contractility in part through mechanisms linked to increased NO production, while TNF and interleukin-1β further promote excessive NO synthesis. Within cardiomyocytes, NO reduces myofibrillar sensitivity to calcium, induces mitochondrial dysfunction, and downregulates *β*-adrenergic receptors, thereby contributing to impaired cardiac performance.

The development of SIMD is multifactorial and involves inflammation, oxidative stress, and apoptosis [18]. NO is generated from l-arginine via NOS activity, with l-citrulline produced as a by-product. Three NOS isoforms – nNOS, iNOS, and eNOS – have been identified in cardiomyocytes. In contrast to iNOS, which is induced by inflammatory stimuli and produces substantial quantities of NO, nNOS and eNOS are constitutively expressed and generate lower levels of NO under physiological conditions. Bougaki et al. demonstrated that activation of eNOS reduced inflammatory cytokine production, attenuated systemic inflammation, and improved myocardial dysfunction in a sepsis model induced by colon ascendens stent peritonitis (CASP) [19]. However, other studies have suggested that eNOS-derived NO may contribute to early myocardial dysfunction during sepsis [20].

Increased iNOS expression has been implicated as a major factor in late-stage cardiac dysfunction associated with sepsis. Excessive NO production via iNOS contributes to alterations in preload and afterload, downregulation of *β*-adrenergic receptors, reduced myofilament responsiveness to Ca^2+^, and mitochondrial dysfunction [20], [21], [22]. Mitochondrial injury is partly mediated by peroxynitrite, a reactive nitrogen species formed through the interaction of NO with superoxide anion, which increases mitochondrial membrane permeability and disrupts cellular energy metabolism [23].

### 3.3 Role of NO in other sepsis-associated organ dysfunction (brain, liver, kidney, lung, and intestine)

Sepsis-associated encephalopathy (SAE) is the most common form of encephalopathy encountered in intensive care settings. It is characterized by diffuse, non-focal cerebral dysfunction that results in acute or chronic cognitive impairment in the absence of direct central nervous system (CNS) infection, structural brain lesions, or alternative causes of encephalopathy, such as hepatic or renal encephalopathy. SAE is defined as diffuse cerebral dysfunction secondary to the systemic inflammatory response to infection, without direct involvement of the CNS. Disturbance of consciousness represents the principal clinical manifestation [24].

Current evidence suggests that the pathogenesis of SAE is largely driven by inflammatory mediators and oxidative stress. These processes contribute to reduced cerebral blood flow, disruption of the blood–brain barrier (BBB), and cerebral edema, ultimately leading to neuronal injury [25]. The BBB is essential for maintaining CNS homeostasis and normal brain function [26]. Its integrity depends primarily on tight junctions between capillary endothelial cells. Disruption of these structures increases BBB permeability, impairs neurovascular unit function, and promotes neuronal damage. Oxidative stress is regarded as a central factor in BBB dysfunction during sepsis. Increased NOS activity activates myosin light-chain kinase, inducing actin–myosin contraction through myosin phosphorylation and leading to disruption of intercellular junctions [26]. This structural alteration compromises endothelial tight junction integrity and contributes to the development of SAE.

Neuronal apoptosis has also been observed in sepsis. Inflammatory mediators, NO, and reactive oxygen species impair mitochondrial function, resulting in reduced adenosine triphosphate (ATP) production, inadequate cellular energy supply, and deoxyribonucleic acid (DNA) damage, which collectively promote neuronal apoptosis.

The liver plays a pivotal regulatory role in sepsis through the production of inflammatory mediators and coagulation factors [27]. Liver dysfunction is common in patients with sepsis and frequently manifests as hyperbilirubinemia and hypoalbuminemia. A relationship between sepsis-associated liver dysfunction and NO has been described. NO directly interferes with hepatic metabolic processes by inhibiting glyceraldehyde-3-phosphate dehydrogenase (GAPDH) and components of the mitochondrial electron transport chain, thereby impairing cellular energy metabolism. In addition, NO activates intracellular guanylate cyclase, increasing cGMP levels. It also suppresses protein synthesis and cytochrome P450 activity, reducing hepatic detoxification capacity and contributing to hyperammonemia. Furthermore, NO-mediated reductions in hepatic perfusion may exacerbate liver injury [28].

Sepsis-induced acute kidney injury (AKI) is a frequent and severe complication associated with high morbidity and mortality. Clinical studies have demonstrated a significant association between excessive iNOS expression and renal tubular injury during systemic inflammatory responses [29]. Endotoxins stimulate iNOS expression in multiple renal cell types, leading to sustained NO production within the kidney. Although NO contributes to vasodilation and participates in processes such as angiogenesis, cell growth, and autophagy, excessive production disrupts renal hemodynamics and promotes tubular injury through peroxynitrite-mediated oxidative stress.

Endothelin-1 (ET-1), an important regulator of renal tubular and vascular function, is also implicated in the pathogenesis of AKI. During sepsis, endogenous NO generated by activated iNOS counterbalances ET-1 overexpression, reflecting a complex regulatory interaction. Activation of matrix metalloproteinase-9 (MMP-9) has been associated with extracellular matrix (ECM) degradation and increased capillary permeability. Evidence suggests that ET-1 and iNOS exert opposing effects on vascular tone, and imbalance between these pathways may contribute to endothelial dysfunction [30].

In sepsis-induced lung injury, neutrophil accumulation and infiltration into pulmonary tissue result in excessive release of inflammatory mediators. This response disrupts pulmonary capillary endothelial function and increases vascular permeability [31]. Excessive NO production by NOS isoforms, together with the formation of peroxynitrite and poly (ADP-ribose), contributes to the development of acute lung injury (ALI) in sepsis. Both nNOS and iNOS participate in pulmonary oxidative and nitrosative stress responses, with nNOS being particularly implicated in the exacerbation of oxidative stress following lung injury and sepsis. Experimental data indicate that genetic deletion of nNOS prevents pulmonary oxidative and nitrosative stress; however, in murine models of sepsis, nNOS deficiency does not improve survival [32]. Early inhibition of nNOS combined with later suppression of iNOS reduces oxidative and nitrosative stress and may improve outcomes in AKI [33].

Sepsis also induces intestinal mucosal injury and barrier dysfunction. Excessive NO production disrupts regulation of intestinal microcirculatory hemodynamics, damages vascular endothelial cells, impairs mucosal defense and metabolic functions, and increases vascular permeability. These alterations facilitate bacterial translocation and contribute to multiple organ dysfunction. Inhibition of excessive NO synthesis attenuates these pathological changes [34], 35].

### 3.4 Impact of NO biochemistry and signaling pathways on organs and systems

Previous investigations have identified three principal inflammatory signaling pathways involved in sepsis: the Janus kinase/signal transducer and activator of transcription (JAK/STAT) pathway, the mitogen-activated protein kinase (MAPK) pathway, and the nuclear factor-κB (NF-κB) pathway. Among these, the JAK/STAT pathway plays a central role in regulating cell proliferation, differentiation, stress responses, inflammation, and immune modulation.

The STAT protein family functions as a key downstream effector of JAK and comprises seven members involved in signal transduction and transcriptional regulation. STAT1 and STAT3 are considered particularly important in inflammatory signaling [36]. Owing to structural differences among STAT proteins, each member regulates transcription of distinct gene subsets [37].

STAT1 was the first member of this family to be characterized. Evidence indicates that IFN-γ activates transcription of interferon regulatory factor-1 (IRF-1) and intercellular adhesion molecule-1 (ICAM-1) via the STAT1 pathway. Phosphorylation of STAT1 has been closely associated with induction of the iNOS gene. *In vitro* studies have shown that inhibition of STAT1 phosphorylation suppresses LPS-induced iNOS expression and decreases NO production [38]. Furthermore, Ohmori et al. reported that IFN-γ-specific antibodies blocked LPS-induced iNOS mRNA expression in macrophages, further supporting the role of STAT1-dependent signaling in iNOS regulation [39].

STAT3 was originally identified as an acute-phase response factor involved in interleukin-6 signaling. Gyurkovska et al. demonstrated that AG490, a tyrosine kinase inhibitor, suppressed STAT3 activity in hepatic tissue from septic mice and consequently reduced iNOS expression. This observation was reinforced by findings that AG490 improved survival in models of LPS-induced septic shock [37]. In addition, ghrelin has been reported to downregulate iNOS expression in pulmonary tissue of septic rats, thereby reducing oxidative stress and attenuating local inflammation [40].

Further evidence suggests that iNOS expression in macrophages is upregulated through activation of NF-κB and Akt signaling pathways [41]. On this basis, it has been proposed that ghrelin-mediated suppression of iNOS expression in septic rats may occur through inhibition of Akt and NF-κB signaling in alveolar macrophages.

### 3.5 Effect of NO on the inflammatory response in sepsis

During sepsis, endotoxins and proinflammatory cytokines induce upregulation of iNOS, resulting in excessive NO production. Elevated NO levels promote marked vasodilation, contributing to hypotension and myocardial dysfunction. Experimental studies have shown that genetic deletion of iNOS in mice prevents endotoxin-induced severe hypotension. In addition, pharmacological inhibition of iNOS activity improves vascular hyporeactivity and ameliorates hypotension in both animal models of septic shock and clinical settings. These findings support the concept that excessive iNOS-derived NO plays a major role in septic vascular hyporesponsiveness and severe hypotension.

Maintaining a physiological balance between iNOS and eNOS may be essential for vascular homeostasis. Sepsis disrupts this equilibrium, contributing to systemic endothelial dysfunction, persistent hypotension, multiple organ dysfunction, and increased mortality.

Multiple studies have demonstrated that NO exerts both detrimental and protective effects. In mammals, NO is synthesized from L-arginine by three NOS isoforms. Under physiological conditions, nNOS and eNOS generate basal levels of NO, which are critical for regulation of vascular tone, neurotransmission, and other homeostatic processes. In contrast, during exposure to LPS and proinflammatory mediators, iNOS produces NO at concentrations that exceed physiological levels, leading to cytotoxic and proinflammatory effects.

Beyond the established contribution of iNOS to inflammation, recent findings suggest a regulatory role for nNOS in inflammatory responses. *In vitro* stimulation of macrophages isolated from septic mice with LPS induces minimal nuclear expression of nNOS. Furthermore, NO-mediated S-nitrosylation of suppressor of cytokine signaling-1 (SOCS1) has been reported to inhibit SOCS1 activity, thereby preventing degradation of DNA-bound NF-κB p65 and sustaining its proinflammatory signaling [41]. These observations underscore the complex and context-dependent regulatory effects of the NOS/NO system in inflammation.

Excessive NO production can also inhibit NF-κB activity through several mechanisms. One mechanism involves S-nitrosylation of NF-κB, which reduces its DNA-binding capacity and transcriptional activity, thereby attenuating inflammatory responses. Additionally, NO may suppress NF-κB activation by inhibiting its nuclear translocation. This process is mediated through S-nitrosylation of IκB kinase (IKK), resulting in reduced IKK activity. Consequently, phosphorylation of IκB decreases, preventing dissociation of the NF-κB dimer from IκB and limiting its translocation to the nucleus.

### 3.6 Modern understanding of sepsis and related therapy

Advances in the understanding of sepsis pathophysiology and clinical manifestations have highlighted the central role of vascular endothelial dysfunction. Under septic conditions, vascular endothelial cells exhibit structural and functional alterations, including pulmonary microcirculatory impairment, endothelial activation, reduction or loss of glycosylated endothelial surface proteins, and disruption of endothelial homeostasis [42]. Microvascular endothelial dysfunction, heterogeneous perfusion, and increased endothelial permeability are considered fundamental mechanisms underlying organ dysfunction and are closely associated with clinical outcomes [43].

Experimental studies indicate that excessive NO production by iNOS during the late stage of sepsis may induce vasodilatory hypothermia and a lethal hypodynamic state. These effects appear to be related to hypothermia and alterations in immune cell migration [44]. Endothelial cells demonstrate organ-specific heterogeneity, and differences between animal and human endothelial responses further complicate interpretation. Under stimulation by inflammatory cytokines or endotoxins, strong induction of iNOS at both mRNA and protein levels has been observed; however, the magnitude and pattern of this response vary across species.

Although numerous animal studies have suggested potential therapeutic strategies targeting NO pathways, cautious interpretation is warranted. Preclinical models may not fully replicate the complex pathophysiology of sepsis in patients, particularly those with comorbidities or receiving multiple concurrent therapies [45]. Consequently, translation of experimental findings into effective clinical interventions remains challenging.

### 3.7 Future directions

Future research should focus on developing regulatory strategies for NO production in sepsis that extend beyond direct inhibition of NOS. Potential approaches include modulation of endogenous regulators of NOS activity, targeted elimination or controlled donation of NO, and optimization of substrate availability [46]. Such strategies may allow more precise adjustment of NO bioavailability while minimizing unintended systemic effects.

Emerging therapeutic directions also include the selective delivery of NO using nanotechnology-based platforms. This approach may help limit excessive NO release during septic shock and reduce reperfusion injury by enabling spatially and temporally controlled NO distribution [47].

Given the dual protective and deleterious roles of NO in sepsis, integrative drug discovery strategies have been explored. A comprehensive virtual screening workflow – incorporating protein–ligand docking, binding free energy calculations, and pharmacokinetic analysis – has been applied to identify candidate compounds targeting endothelial soluble guanylate cyclase. Such compounds may offer therapeutic benefit in sepsis-associated vascular paralysis characterized by excessive vasodilation [48].

## 4 Conclusions

NO exerts a complex and isoform-dependent influence on the pathophysiology of sepsis. Excessive NO production, particularly derived from iNOS, is strongly associated with vascular dysfunction, circulatory shock, and multiple organ failure. In contrast, NO generated by eNOS may confer protective effects, particularly during the early stages of sepsis.

This duality highlights the clinical importance of isoform-specific modulation of NO signaling – limiting excessive iNOS activity while preserving or potentially enhancing eNOS-mediated physiological functions. Future investigations should emphasize the development of highly selective iNOS inhibitors and the refinement of patient stratification strategies based on biomarkers, such as circulating LPS levels or tissue-specific iNOS expression.

Integration of biomarker-guided stratification with targeted therapeutic interventions may improve treatment precision, reduce unintended systemic effects, and ultimately enhance clinical outcomes. Early recognition of sepsis, accurate assessment of disease severity, and timely therapeutic intervention remain fundamental to reducing sepsis-related morbidity and mortality.
